# Do nitric oxide, carbon monoxide and hydrogen sulfide really qualify as ‘gasotransmitters’ in bacteria?

**DOI:** 10.1042/BST20170311

**Published:** 2018-09-06

**Authors:** Lauren K. Wareham, Hannah M. Southam, Robert K. Poole

**Affiliations:** 1 Vanderbilt University Medical Center, Vanderbilt Eye Institute, 11420 Medical Research Building IV, 215B Garland Avenue, Nashville, TN 37232-0654, U.S.A.; 2 Department of Molecular Biology and Biotechnology, The University of Sheffield, Firth Court, Sheffield S10 2TN, U.K.

**Keywords:** carbon monoxide, cellular signalling, gasotransmitters, hydrogen sulfide, nitric oxide

## Abstract

A gasotransmitter is defined as a small, generally reactive, gaseous molecule that, in solution, is generated endogenously in an organism and exerts important signalling roles. It is noteworthy that these molecules are also toxic and antimicrobial. We ask: is this definition of a gasotransmitter appropriate in the cases of nitric oxide, carbon monoxide and hydrogen sulfide (H_2_S) in microbes? Recent advances show that, not only do bacteria synthesise each of these gases, but the molecules also have important signalling or messenger roles in addition to their toxic effects. However, strict application of the criteria proposed for a gasotransmitter leads us to conclude that the term ‘small molecule signalling agent’, as proposed by Fukuto and others, is preferable terminology.

## Introduction: Don't shoot the messenger!

The term ‘gasotransmitter’ or ‘gaseous transmitter’ appears to have been coined ca. 2002 [1] to describe endogenously generated gaseous molecules that play important physiological roles. Specifically, they were defined [1,2] by the following six criteria:

(i) They are small (molecular mass 28–34) molecules of gas [such as nitric oxide (NO), carbon monoxide (CO) and hydrogen sulfide (H_2_S)].
(ii) They are freely permeable through membranes and therefore act without cognate membrane receptors. In this respect, they differ from better-established transmitters such as hormones, neurotransmitters and drugs.
(iii) Their endogenous generation is enzymatic and highly regulated, a characteristic that chimes with the potentially toxic nature of the best established of the gasotransmitters — currently NO, CO and H_2_S.
(iv) They have well-defined, specific functions at physiologically relevant, generally exceptionally low, concentrations. Manipulating endogenous levels evokes specific physiological changes.
(v) Their functions can be mimicked by exogenous counterparts (for example, NO-releasing compounds or CO-releasing molecules, CORMs)
(vi) Their cellular effects may or may not be mediated by secondary messengers, but specific final targets are implicated. For example, NO may result in vasodilation by prior interaction with soluble guanylate cyclase (GC, formerly sGC), or may bind directly to haem proteins and a multitude of other targets.

However, as observed by Samuel Butler, ‘Definitions are a kind of scratching and generally leave a sore place more sore than it was before’. In this short review, we do not wish to scratch too vigorously, but feel that, in microbial science in particular, it is useful to question the early definitions. The term has been widely adopted: a recent Web of Science search reveals almost 1200 hits. A major criticism of the term is that each of these three molecules is completely soluble at physiologically relevant concentrations: the term ‘gas’ does not accurately describe their physical state or chemical reactivity [3]. Nevertheless, one uniting feature lies in their biochemical targets, i.e. the reactive centres with which they interact (redox-active metals or amino acids, notably cysteine thiols or tyrosine phenols). The commonality of targets disguises, however, the distinctions in reaction mechanisms that underpin the ability of ‘gasotransmitters’ to achieve exquisite control.

Here, we consider how relevant and precise the term ‘gasotransmitter’ is when applied to bacteria [4,5]. To what extent are the above criteria met? We must question, for example, the nature of the signal (if any) being transmitted by the molecule and its targets. Is the term ‘small molecule signalling agent’ proposed before [3] more apt in microbes? Note that we do not detail those chemical species that are chemically related to the gases NO, CO and H_2_S. For example, the oxidation/reduction products of NO, carbonylation reactions that introduce CO onto organic and inorganic substrates and the multiple low-molecular-mass persulfides are discussed only *en passant*. The topic has been reviewed on numerous occasions before, including in this journal [6] and *The Biochemist* (e.g. [7–9]). New members of the gasotransmitter family are proposed, such as sulfur dioxide [10].

## The eukaryotic exemplars: NO, CO and H_2_S

This trinity comprises the first ‘gasotransmitters’ to be identified and they remain the best studied. Since its discovery as a signal mediator in the early 1980s, NO has been implicated in a myriad of physiological processes with roles in the immune, cardiovascular and nervous systems [11]. Nitric oxide synthase (NOS) is the enzyme responsible for endogenous NO production and three isoforms exist in mammals: the constitutive forms: neuronal NOS1 (nNOS) and endothelial NOS3 (eNOS), and the inducible NOS2 (iNOS). NOS enzymes were initially classified by their localisation and expression patterns, but this is misleading: eNOS is found in cells and tissues other than the endothelium, nNOS is found in cells other than neurons and iNOS is found constitutively in some tissues [12]. Nevertheless, the diverse expression and subcellular locations of NOS leads to an array of NO-mediated physiological effects.

Whereas NO was discovered as the chemical mediator of an important biological function (most famously vasodilation), the production of CO in mammalian physiology was recognised long before much attention had been devoted to its biological function [13]. Its presence in biology was thought to be merely as a waste product of the degradation of haem, a reaction catalysed by the enzyme haem oxygenase (HO), also liberating Fe and biliverdin. However, CO has latterly been recognised as a potent biological messenger in mammalian systems. Haem oxygenases exist in constitutive (HO-2) and inducible (HO-1) isoforms [14]. While HO-2 is predominantly expressed in testes, brain and the endothelium, HO-1 is up-regulated in tissues following various stress stimuli, including oxidative stress, which is an underlying factor in different pathological states [15]. At physiological levels, CO has been shown to mediate a broad array of signalling processes, including the production of inflammatory mediators [16], cell survival and apoptosis [17], signalling in the central nervous system [18] and in bacterial infection [19].

By comparison, the acceptance of H_2_S as a gasotransmitter is more recent. Although the presence of H_2_S in tissue had been noted for decades, and its potential signalling capacity was first noted in the late 1990s [20], the argument for gasotransmitter status, founded on its endogenous metabolism and physiological functions, was made much later [1]. H_2_S is endogenously generated via both non-enzymatic and enzymatic pathways. In mammalian tissues, the enzymatic production of H_2_S from l-cysteine is catalysed by cystathionine-β-synthase (CBS), cystathionine-γ-synthase (CSE) and the 3-mercaptopyruvate sulfurtransferase/cysteine aminotransferase (3-MST/CAT) pathways (e.g. [21,22]). Like NO and CO, H_2_S at very low levels affects human physiology at molecular, cellular, tissue and system levels [22,23].

Thus, gasotransmitters in mammalian systems are endogenous signalling molecules; cellular production leads to their binding to cell surface receptors, intracellular receptors and proteins, triggering a cascade of events that lead to multiple physiological outcomes. Are these effects mirrored in microbes?

## NO in microbes: gasotransmitter or not?

The products of NO oxidation by oxygen (NO_2_), the nitrosonium cation (NO^+^), the nitroxyl anion (NO^−^) and peroxynitrite (ONOO^−^; the product of the reaction of NO with superoxide) are outside the scope of this short review. As we have argued before [24,25], although these congeners of NO are of immense biological importance, and are ‘close cousins’, they are not NO. That NO satisfies criteria (i) and (ii), namely in being a small molecule that permeates membranes, is evident, but what is the origin of NO in microbes?

**Figure 1.**
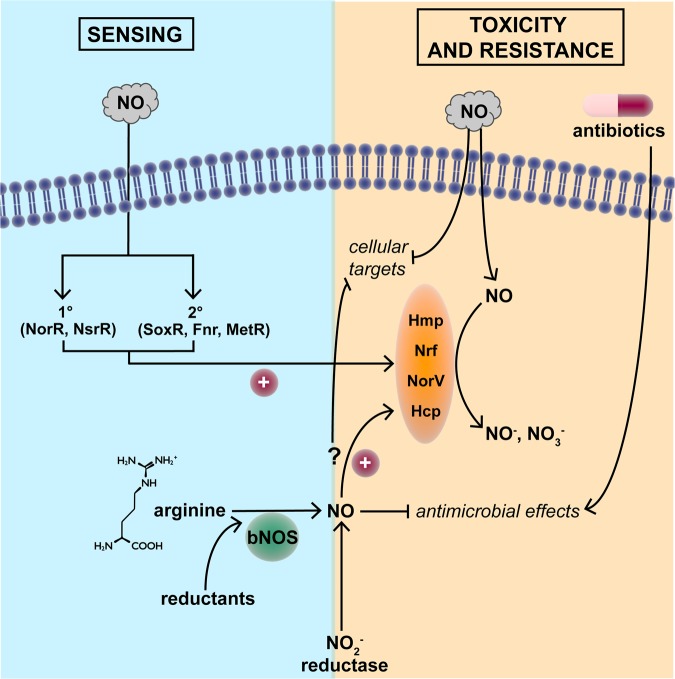
NO sensing and mechanisms of resistance to toxicity. NO readily enters microbial cells from diverse exogenous sources, but may also be synthesised from arginine by bacterial NO synthase (bNOS). Primary and secondary sensors detect NO, which exerts numerous antimicrobial effects, but paradoxically may protect bacteria from antibiotic action. The toxicity of NO is minimised by NO-detoxifying proteins (Hmp, Nrf, NorV and Hcp). However, it is unclear whether endogenously generated NO elicits equivalent defence responses. For details, see the text.

One criterion for a gasotransmitter is that it should be endogenously generated. The most significant sources of NO in a bacterial context are (a) synthesis by NOS in the gut epithelium, (b) release by denitrifying bacteria and nitrite reduction [26] and (c) synthesis by iNOS [27] as a component of the antibacterial weaponry in phagocytic cells (macrophages and monocytes) [28] on stimulation by cytokines. Minor sources include anaerobic reduction of nitrite to ammonia or the chemical reduction of nitrite by FeS proteins or haemproteins [29]. However, bacteria also possess NOS enzymes (bacterial NOS, bNOS; Figure 1) that share similarities with the mammalian forms [27], but lack the reductase domain of eukaryotes. Electron donation is achieved from specialist or promiscuous cellular reductases [30,31]. Specialised roles for bacterial NOS have been proposed in nitration reactions [32], resistance to oxidative stress and antibiotics [33,34], and host colonisation [35].

Further criteria (iv–vi) are that the small molecule should have well-defined and specific functions at physiologically relevant concentrations and that its levels should evoke specific physiological changes. A clear example of a function for bNOS-generated NO is its role as a virulence factor in *Bacillus anthracis* [36], in which NO-mediated activation of bacterial catalase and suppression of the damaging Fenton reaction protects the bacterium from the immune oxidative burst. More striking is the role of NO from bNOS in increasing the resistance to antibiotics [34]. This appears to be achieved in two ways: chemical modification of the drugs (e.g. nitrosation reactions with arylamino moiety of acriflavin) and alleviating the impact of oxidative stress that is proposed to accompany antibiotic action. Certain bacteria exploit bNOS-derived NO in competition with others: *Bacillus* sp. use NO to reduce the oxidative stress associated with pyocyanin production by *Pseudomonas* [34]. However, many aspects of their functions, particularly in the NO-producing cell, remain obscure.

With such exceptions, it is unclear what *function* NO fulfils in bacteria; this is for semantic debate, perhaps. However, if we accept that the function of intracellular NO may be to signal the presence of *exogenous* NO as a potentially toxic agent, then the sensing mechanism(s) are relevant.

## NO sensing and signalling

An interesting example of NO acting as a signal between bacterium and host is that of *Moraxella catarrhalis* [37]. The nitrite reductase NirK (also known as AniA) generates NO and it is thought that this diffuses into the proximate environment of the bacterium where it increases the secretion of tumour necrosis factor alpha and modulates the expression of apoptotic proteins, therefore triggering host cell programmed death, thus dysregulating host cell gene expression. Conversely, nitrite-derived NO is toxic towards *M. catarrhalis* in maturing biofilms [37,38]. An opposite case might be the consumption of NO by meningococci [39]. We demonstrated that expression of meningococcal NorB increased the rate at which low-molecular-mass *S*-nitrosothiols (SNO) decompose *in vitro*. We then induced SNO formation in murine macrophages by activation with lipopolysaccharide and γ-interferon, resulting in a reduced abundance of SNO during co-incubation with *Neisseria meningitidis*, *Salmonella enterica* or *Escherichia coli.* In each case, this was dependent on bacterial NO detoxification genes, which prevented SNO formation through the removal of NO. This may represent a novel mechanism of NO-mediated host cell injury by bacteria.

NO sensors (Figure 1) may be subdivided into those that are specialised (or dedicated), and those that are secondary, in the sense that the sensor responds to another, primary signal. The prototypical secondary sensor is SoxR, its primary function being sensing of superoxide anion by virtue of a reactive [2Fe-2S] cluster that becomes oxidised, presumably by superoxide itself [40]. However, NO also activates SoxR and induces expression of genes in the SoxRS regulon. A further example is the bacterial redox sensor Fnr: its primary signal is O_2_, which reacts with the [4Fe-4S], via a mechanism that has been the subject of intensive studies [41]. However, Fnr also senses NO [42]. The Fnr homologue in *Azotobacter vinelandii*, CydR, is also both oxygen- and NO-reactive [43]. Subsequently, many other bacterial FeS proteins were reported to sense NO, e.g. the NO-responsive regulator of flavohaemoglobin gene expression in *Streptomyces coelicolor*[44]. Other secondary sensors include OxyR, MetR, DosS and Fur (for a review, see ref. [45]).

A well-established example of a dedicated NO sensor is that of NorR. In *E. coli*, NorR activates the divergently transcribed *norVW* genes in response to NO (or nitroprusside, GSNO or acidified nitrite, which we do not discuss here; see ref. [25]). These NorR-activated genes encode a flavorubredoxin and its redox partner that reduce NO to nitrous oxide at the expense of NADH [46–48]. NorR is activated by the formation of a mono-nitrosyl complex at a mononuclear iron centre (not an [FeS] cluster or haem) in the GAF domain [49,50]. Ferrous NorR reacts with NO *in vitro*, suggesting that NorR is a sensor of NO *per se*. However, in other bacteria, as in *Pseudomonas aeruginosa*, NorR activates transcription of the *fhp* gene (encoding flavohaemoglobin) in response to NO [51]. Thus, NorR is a true NO sensor and turns on genes exclusively involved in NO metabolism, but the physiological cue signalled by this mechanism in nature is unclear.

NsrR is also an NO-responsive regulator in many bacteria including *E. coli* [52,53], *Bacillus subtilis* [54], *Neisseria gonorrhoae*[55] and *S. coelicolor* [56]. Numerous genes may be so regulated but in *S. coelicolor* only two flavohaemoglobin genes (*hmpA1* and *hmpA2*) are targets. These genes encode the NO-detoxifying flavohaemoglobin, rationalising the control exerted. The *S. coelicolor* NsrR contains an NO-reactive [4Fe-4S] cluster and exhibits high-affinity DNA binding to the operators of the NsrR-regulated genes. The reactions with NO are complex with up to 8–10 NO molecules reacting per cluster, leading to the formation of several iron-nitrosyl species [44].

Distinct classes of NO sensors are represented by the H-NOX proteins (haem-NO or oxygen-binding domain) [57,58] and the NosP protein discovered by Boon and colleagues. These haem proteins are widely distributed in bacterial genera and encoded in operons with genes for kinases or cyclic di-AMP synthesis/phosphodiesterase enzymes. In *P. aeruginosa*, it is well recognised that NO ‘directs’ the bacterium to disperse biofilms at picomolar concentrations and several proteins have been implicated, namely BdlA, DipA and others (see [58]). NosP is in the same operon as a gene for kinase previously implicated in biofilm formation. Biofilms of a mutant in the NosP signalling pathway lose the ability to disperse in response to NO [57], so NosP may be a global biofilm regulator. In *Vibrio cholerae*, NO binding to NosP inhibits the autophosphorylation activity of a histidine kinase implicated in quorum sensing. In other words, NO seems to regulate quorum sensing and biofilm formation, and may be a clear case of NO acting as a signal that alters bacterial behaviour. Beneficial effects of NO signalling are also reported in yeast [59]. However, why such physiological responses should be controlled by NO or what the source of that NO might be in natural environments is less clear.

## CO sensing in microbes?

Unlike NO, CO has no obvious function in the vast majority of bacteria, with a few notable exceptions. Some prokaryotes that survive in hostile conditions sense and metabolise CO [60,61]. Often, these sensing proteins bear a haem cofactor that provides an allosteric binding site for CO due to the high affinity of the iron (witness the binding of CO to globin proteins). The most well-studied haem-dependent gas-sensing transcription factor is CooA (CO oxidation activator) from *Rhodospirillum rubrum* [62,63]. CooA, a member of the FNR/CRP superfamily of transcriptional regulators, is a homodimeric protein comprising an N-terminal haem-binding domain and a C-terminal helix–turn–helix DNA-binding domain. The haem iron undergoes a redox-mediated ligand switch as the mechanism for activation. A further haem-containing transcription factor associated with CO metabolism occurs in *Burkholderia xenovorans* (LB400) [64], which catalyses dissimilatory nitrate reduction and aerobic CO oxidation [65]. The *cox* genes that encode the molybdo-flavoprotein complex required for CO oxidation are regulated by the transcription factors RcoM-1 and RcoM-2 (regulator of CO metabolism). In this sense, then, CO may be considered as a ‘transmitter’, the signal being the availability of a useable, if unlikely, carbon source. It has been suggested that CO, occurring at a concentration of ∼700 ppm on Mars, could support near-surface bacterial activity; an abundant potential oxidant is perchlorate [66].

A more familiar part played by CO in biology is that of poison. CO is a notoriously toxic gas, binding primarily to ferrous oxygen-reactive haem proteins [67] in competition with oxygen. CO is generally considered to be toxic to microorganisms. It is used to preserve meat [68], but many bacteria are relatively resistant, in part, because they possess CO-insensitive oxidases, such as cytochrome *bd* [69]. Indeed, airborne bacteria survive high urban CO concentrations [70], and bacterial cultures may be bubbled with CO with little toxicity [71,72].

**Figure 2.**
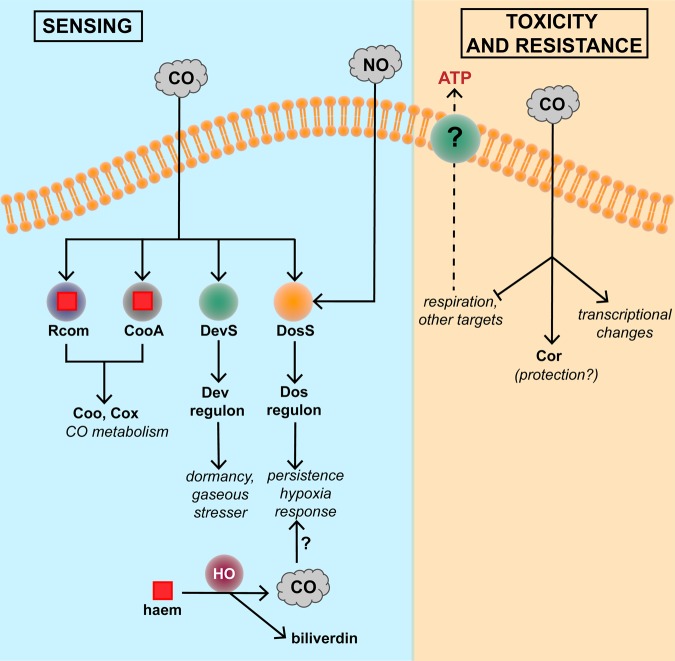
Carbon monoxide sensing and mechanisms of resistance to toxicity. CO readily enters microbial cells from diverse exogenous sources, but may also be synthesised by endogenous haem oxygenase activity; the role of endogenously derived CO is unclear. Numerous sensors detect CO, leading to CO metabolism or downstream gene regulation. CO exerts numerous antimicrobial effects, but Cor is reported to afford protection. The reported effects of CO on ATP release from bacteria are unexplained. For details, see the text.

As described above, CO is generated endogenously in mammals by HO-catalysed breakdown of haem and, although relatively inert from a chemical standpoint, CO is an established gasotransmitter. Certain bacteria (e.g. *Mycobacterium tuberculosis*) sense host-derived CO (Figure 2). The DevRS/DosT (also called DosRS/DosT) system regulates an ∼50-member regulon in response to hypoxia, NO and CO [73–75]. More recently, the DevR regulon has been implicated in the transition of *M. tuberculosis* from an actively respiring to a latent non-replicating persistent state [76].

Curiously, infection with *M. tuberculosis* induces (and co-localises with) HO1 in both mouse and human tuberculosis lesions and primary human macrophages. This induction itself appears to be a virulence mechanism since experimental inhibition of HO1 reduces inflammatory cytokine production and restricts mycobacterial growth [77]. It is unclear whether HO1 is beneficial or detrimental during human infections by mycobacteria.

Wegiel et al. [71] recently hypothesised that bacteria exposed to CO release ATP (Figure 2), which activates inflammatory pathways. This appears to be a clear example of a gasotransmitter role, albeit between species: it is proposed that HO-1 is induced by endotoxin (LPS) (from bacteria) and generates CO. CO emitted by the host macrophage ‘compels the bacteria to generate ATP’, which acts as a danger-associated molecular pattern for the macrophage. The bacteria-derived ATP binds to a purinergic receptor on the macrophage, leading to activation of the inflammasome to drive pathogen clearance. There are several puzzling aspects of this claim. First, we are unaware of any extracellular or periplasmic location of ATP in bacteria, implying that the ATP ‘leaked’ from damaged cells, although the authors state that bacteria were viable. Second, the ATP release was attributed to a CO-dependent *increase* in bacterial ATP levels, a finding hard to reconcile with inhibition of respiration by CO.

If CO is toxic, it might be expected that bacteria could possess genes that confer CO resistance. One such gene, *cor*, has been described in *M. tuberculosis* (Figure 2). Zacharia et al. [78] screened an *M. tuberculosis* transposon library for mutants susceptible to an atmosphere of 2% CO and found that disruption of Rv1829 (carbon monoxide resistance, Cor) led to marked CO sensitivity. Heterologous expression of Cor in *E. coli* rescued it from CO toxicity, although this assay used CORM-2 [tricarbonyldichlororuthenium(II) dimer] as a CO source, not CO itself. The impact of the redox-active ruthenium ion [79] should be assessed in further studies. Importantly, the virulence of the *cor* mutant was attenuated in a mouse model of tuberculosis. Thus, Cor appears to protect bacteria from host-derived CO. Conceivably, this example could serve as an illustration of a bacterium receiving a signal on the presence of CO, but the molecular mechanisms of the CO sensing and its detoxification are unknown.

## H_2_S sensing and signalling in microbes

H_2_S is emerging as an important mediator or gasotransmitter, promoting the resolution of inflammation and injury repair [22,80]. A recent bibliometric analysis of publications in biology and medicine shows almost 6000 papers between 1990 and 2016 [81]. Although the term ‘hydrogen sulfide’ is most commonly used [20], H_2_S is a weak acid in aqueous solutions — dissociating into its anions HS^−^ and S^2−^. As the dissociation equilibrium between H_2_S and HS^−^ is at pH ∼7, in physiological conditions ‘H_2_S’ exists predominantly as its anion HS^−^ (∼80%), H_2_S (∼20%) and negligible amounts of S^2−^, which are collectively termed the ‘sulfide pool’ [82]. Thus, although it has not been possible to determine which form of H_2_S (H_2_S, HS^−^ or S^2−^) is active, the term ‘hydrogen sulfide’ has been used in toxicity studies [20], and it is customarily regarded as a *gas*otransmitter [23].

**Figure 3.**
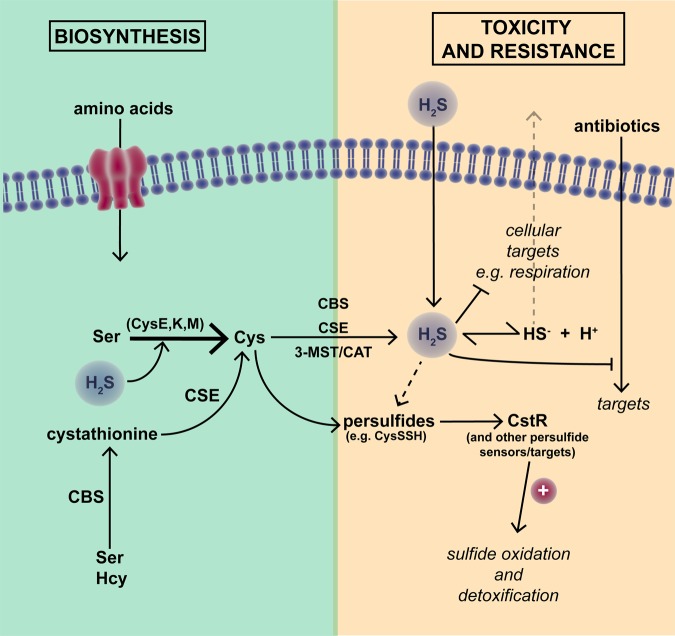
Hydrogen sulfide biosynthesis, signalling and regulation. As described in the text, H_2_S is synthesised by diverse pathways from cysteine and cystathionine, but also readily permeates membranes in the unionised form. Specific H_2_S synthesis enzymes include cystathionine-β-synthase (CBS), cystathionine-γ-synthase (CSE) and the 3-mercaptopyruvate sulfurtransferase/cysteine aminotransferase (3-MST/CAT) pathway. Persulfides (e.g. CysSSR) are key to H_2_S signalling pathways. CstR is a persulfide-sensing sulfide stress-response regulator in *S. aureus*.

The principal pathways that mediate H_2_S production in mammalian systems (e.g. [21,22]) are shown in Figure 3. A more recently discovered route involves 3-MST and d-amino acid oxidase [83]. H_2_S in higher organisms also arises in part from bacterial sulfur metabolism. Homologues of CBS, CSE and 3-MST/CAT also appear to be ubiquitous across bacterial genomes [84], although in most bacteria l-cysteine is produced enzymatically (via CysE, CysK and CysM) from l-serine. For a review, see [85]. H_2_S may also be generated in microbes by other physiological processes, i.e. via degradation of cysteine and other sulfur-containing amino acids/peptides, or non-enzymatically, by dissimilatory reduction of inorganic sulfur compounds [84,86] (Figure 3). Non-enzymatic generation of H_2_S by obligate or facultative anaerobic sulfate-reducing bacteria occurs via the utilisation of inorganic sulfur compounds such as sulfates, sulfites or polysulfides as alternative terminal electron acceptors for reduced organic substrates [86].

H_2_S, like NO but unlike CO, is reactive. Its signalling effects are complex since it interacts with oxygen and NO. These reaction products are outside the scope of this review. At elevated concentrations, extracellular H_2_S is toxic to bacteria, complexing with transition metals, leading to respiratory inhibition and covalent modification of haem-containing and Fe–S containing enzymes [82]. Recently, a specific terminal oxidase, cytochrome *bd*, has been shown to be required for sulfide tolerance [87]; the same oxidase also confers tolerance to NO [88] and CORM-2 [69]. Although H_2_S-induced toxicity is linked to oxidative damage via inhibition of antioxidant enzymes [89], low levels of intracellular H_2_S protect against metal ion toxicity, antibiotics and oxidative damage. H_2_S directly scavenges metals ions and reactive oxygen species (ROS) and prevents Fenton chemistry and related antibiotic-induced oxidative damage [82,84,90]. H_2_S-mediated signalling processes appear to play an important role in microbial resistance to oxidative stress [82,91].

Low-molecular-mass persulfides such as cysteine hydropersulfide (CysSSH) and glutathione (GSSG) have long been recognised as important metabolites, and recent advances in analytical tools have identified many such species in prokaryotes and eukaryotes [85,92], but these are outside the scope of this review. H_2_S signalling occurs via post-translational modification of protein cysteine residues (–SH groups) to form the persulfide moiety (RSSH), which in turn modulates protein activity [91]. This process (persulfidation or ‘S*-*sulfhydration’) is the major mechanism of H_2_S signalling in mammalian systems leading to the classification of H_2_S as the third gasotransmitter. In evolutionarily diverse bacteria, sensing of persulfides typically involves transcriptional repressors that contain conserved cysteine residues that regulate DNA-binding activity. Persulfidation or oxidation of these residues induces structural changes leading to derepression of sulfide oxidation genes when reactive sulfide species (RSS) are present [93]. Proteomic profiling of *Staphylococcus aureus* in response to exogenous sulfide revealed that persulfidation regulates many key metabolic enzymes and ROS and RNS stress-response systems and other virulence-related genes, including the persulfide stress-response regulator CstR [91]. In addition, there appears to be a dedicated thioredoxin-mediated hydrosulfide reduction system that functions to reverse persulfidation in *S. aureus* [91,94].

To what extent is H_2_S a true signalling effector molecule in microbes? Though it is clear that bacteria both produce and sense H_2_S, albeit indirectly via related RSS, it is currently unclear whether endogenously produced H_2_S is an effector molecule that induces a signalling response in the same bacterial cell or if H_2_S-mediated responses are more a response to environmental and/or host-derived H_2_S or RSS.

## Conclusions

There can be no doubt that in microbes, as in higher organisms, small molecules that can exist as gases (‘gasotransmitters’) are both generated and sensed. In the bacterial world, an extraordinarily sophisticated body of knowledge has been acquired on the generation of these signals/molecules and on their perception. However, in very few cases, do we understand what the signals *mean*. Many bacteria respond, for example, to NO, but why? The clearest rationalisation may be the protective response of bacteria that have been engulfed by phagocytic cells. Strictly adopting the criteria laid down in eukaryotic cells leads us to the view that, in such a case, the gas molecule is not a *transmitter*, being not generated endogenously (i.e. within the bacterium). Conversely, why do bacteria make NO? Similar doubts are raised regarding CO and H_2_S: to be regarded as a true ‘gasotransmitter’, the small signalling molecule should derive from, and be active in, the same cell/organism. Because many aspects of the microbial effects of these gases remain poorly resolved, it is perhaps dangerous to adopt the term ‘gasotransmitter’, which has been so precisely defined (Table 1). This leads us to conclude that the term ‘small molecule signalling agent’, as proposed by others, is preferable terminology in the case of bacteria.

**Table 1:** Do NO, CO and H_2_S qualify as gasotransmitters? For criteria (iv) to (vi), examples are given.

Criterion	NO	CO	H_2_S
(i) Small gas	✓	✓	✓
(ii) Membrane-permeable	✓	✓	✓
(iii) Endogenously synthesised	✓	✓	✓
(iv) Specific functions; manipulating endogenous levels evokes changes	✓ antibiotic resistance, virulence, quorum sensing, biofilms, etc.	unclear, except in CO metabolism^1^	✓ antibiotic resistance, oxidative stress resistance
(v) Effects mimicked by exogenous counterparts	✓	Only toxicity can be mimicked by CO or CORMs?	✓
(vi) Specific cellular/molecular targets^2^	✓	unclear	Only via persulfide chemistry

1 We do not regard use of CO as a metabolic substrate as signal transduction or gasotransmitter functions; endogenous CO is not used in this way.

2 We exclude targets such as metal centres that may lead to toxicity.

Conductors must give unmistakable and suggestive signals to the orchestra — not choreography to the audience

(George Szell, conductor, 1897–1970)

## Abbreviations

3-MST: 3-mercaptopyruvate sulfurtransferase
CAT: cysteine aminotransferase
CBS: cystathionine-β-synthase
CO: carbon monoxide
CooA: CO oxidation activator
Cor: carbon monoxide resistance
CORMs: CO-releasing molecules
CSE: cystathionine-γ-synthase
eNOS: endothelial NOS
H_2_S: hydrogen sulfide
Hcy: homocysteine
HO: haem oxygenase
iNOS: inducible NOS
nNOS: neuronal NOS
NO: nitric oxide
NOS: Nitric oxide synthase
ROS: reactive oxygen species
SNO: *S*-nitrosothiols
